# Cancer-Associated Fibroblast-Derived Interleukin-8 Promotes Ovarian Cancer Cell Stemness and Malignancy Through the Notch3-Mediated Signaling

**DOI:** 10.3389/fcell.2021.684505

**Published:** 2021-07-01

**Authors:** Zhaodong Ji, Wenjuan Tian, Wen Gao, Rongyu Zang, Huaying Wang, Gong Yang

**Affiliations:** ^1^Cancer Institute, Fudan University Shanghai Cancer Center, Shanghai, China; ^2^Department of Oncology, Shanghai Medical College, Fudan University, Shanghai, China; ^3^Gynecological Oncology, Fudan University Shanghai Cancer Center, Shanghai, China; ^4^The Cancer Hospital of the University of Chinese Academy of Sciences (Zhejiang Cancer Hospital), Institute of Basic Medicine and Cancer (IBMC), Chinese Academy of Sciences, Hangzhou, China; ^5^Ovarian Cancer Program, Division of Gynecologic Oncology, Department of Obstetrics and Gynecology, Zhongshan Hospital, Fudan University, Shanghai, China; ^6^Central Laboratory, The Fifth People’s Hospital of Shanghai, Fudan University, Shanghai, China

**Keywords:** IL-8, tumor stemness, epithelial ovarian cancer, cancer-associated ovarian fibroblasts, Notch3 signal pathway

## Abstract

As a significant component in ovarian cancer microenvironment, cancer-associated fibroblasts (CAFs) contribute to cancer progression through interaction with cancer cells. Recent studies demonstrate that interleukin-8 (IL-8) is overexpressed in multiple cancer types and is essential for tumor development. Nonetheless, the underlying mechanism that the CAF-derived IL-8 promotes ovarian tumorigenesis is unknown. Here, we show that IL-8 secreted from CAFs could activate normal ovarian fibroblasts (NFs) through multiple signaling and that IL-8 stimulated malignant growth of ovarian cancer cells in animals and increased the IC_50_ of cisplatin (CDDP) in ovarian cancer cells. Further study showed that IL-8 induced cancer cell stemness via the activation of Notch3 and that the high level of IL-8 in ascites was positively correlated with the expression of Notch3 in ovarian cancer tissues. Collectively, IL-8 secreted from CAFs and cancer cells promotes stemness in human ovarian cancer via the activation of the Notch3-mediated signaling, which may provide a novel strategy for ovarian cancer treatment.

## Introduction

Ovarian cancer is the second most common cause of gynecologic malignancies around the world, with a high rate of metastatic recurrence and chemoresistance after first surgery (Eisenhauer, 2017). Over the past decades, most studies have been focused only on characteristics of cancer cells rather than the tumor stroma. A growing number of evidence suggests that the tumor microenvironment not only contributes to the initiation and development of malignancy but also promotes cancer metastasis and recurrence, while the secreted chemokines including interleukin (IL)-6 and IL-8 may play vital roles in ovarian tumorigenesis (Browning et al., 2018). In ovarian cancer microenvironment, IL-8, as an adipokine, can activate adipocytes along with the fatty acid-binding protein 4 (FABP4) to provide fatty acids, which provides energy to promote cancer cell omental metastasis (Nieman et al., 2011). Cancer-associated fibroblasts (CAFs) also stimulate omental metastasis of cancer cell through TGF-β-activated of MMP-2 (Cai et al., 2012). However, the molecular mechanism that IL-8 mediates the stroma–cancer interaction to promote the growth and metastasis of ovarian cancer is still unclear.

Cellular senescence is an irreversible phenomenon that cells stop to divide due to shortened telomere. Senescent fibroblast is an important component of stromal cells in the tumor microenvironment with unique chracteristics (Wang et al., 2017). In the process of culture, they become morphologically flattened with the elevated activity of senescence-related acidic β-galactosidase. Many inflammatory cytokines including IL-8 can be secreted by senescent fibroblasts to promote tumorigenesis and metastasis (Krtolica et al., 2001; Orjalo et al., 2009; Lee et al., 2012). Our team previously found that the growth-regulated oncogene 1 (Gro-1) secreted from CAFs promoted epithelial ovarian tumorigenesis (Yang et al., 2006). However, the underlying mechanism that cytokines secreted from CAFs activate normal ovarian fibroblasts (NFs) and thereby promote epithelial malignancy remains unclear.

The Notch family genes were first identified in 1983 (Kidd et al., 1983) and reported to be involved in multiple functions including cancer stem cell (CSC) self-renewal, cancer angiogenesis, metastasis, recurrence, and chemoresistance (Miele et al., 2006; Purow, 2012). Activation of Notch signaling pathway in bone stroma enhances bone metastasis of breast cancer (Sethi et al., 2011). In ovarian cancer, Notch3 and Notch1 usually activate multiple signaling pathways to participate in cancer development (Park et al., 2006; Rose et al., 2010). Overexpression of Notch3 is associated with ovarian cancer recurrence and chemoresistance to carboplatin (Park et al., 2010), which predicts a poor prognosis in ovarian cancer (Jung et al., 2010). Overexpression of Notch3 also enriches ovarian cancer cells with stem-like cell properties, leading to chemoresistance to platinum-based therapy (McAuliffe et al., 2012). Jagged 1, as a Notch ligand, could promote angiogenesis in endothelial cells and lead to proliferation and chemoresistance in epithelial cancer cells (Steg et al., 2011). In addition, Notch/Delta-like ligand 4 (DII4) also functions in both tumor and endothelial cells, which could stimulate ovarian cancer growth (Hu et al., 2011). Blocking of the Notch1 activity by inhibiting gamma-secretase is unable to release Notch intracellular domain (NICD), which retards ovarian tumor growth and induces ovarian cancer cell apoptosis (Wang et al., 2010). Recent studies also revealed that the Notch signaling activity may regulate a network of inflammatory cytokines in ovarian tumor microenvironment (Kulbe et al., 2012; Wieland et al., 2017).

In the present study, we found that IL-8 derived from CAFs could promote angiogenesis and proliferation of NFs through the activation of the AKT and ERK pathways and could induce the stemness and malignant proliferation of ovarian cancer cells through the Notch3-mediated signaling.

## Materials and Methods

### Cell Lines and Cell Culture

NFs (NF320 and NF325) and cancer-associated ovarian fibroblasts (CAF501 and CAF502) were isolated from ovarian tissues with informed consent of the donors according to a protocol approved by the Institutional Review Board and were cultured for this study at early population doublings according to the method described elsewhere (Rosen et al., 2006). Human epithelial ovarian cancer (EOC) cell line HEY-A8 was purchased from American Type Culture Collection (Manassas, VA, United States) and cultured in RPMI-1640 media containing 10% fetal calf serum, 2 mM of L-glutamine, penicillin (100 units/ml), and streptomycin (100 μg/ml). Ovarian cancer cell line HEY-A8 was infected with Notch3 report plasmid with green fluorescent protein (GFP). Ovarian cancer cell line HEY-A8 was infected with IL-8 cDNA and IL-8 shRNA viruses with anti-puro.

### Measurement of Interleukin-8 Secretion

The levels of IL-8 in conditioned media (CM) from NFs and CAFs were measured by enzyme-linked immunosorbent assay (ELISA) Quantikine kits from R&D Systems (Minneapolis, MN, United States) (DGR00 and D8000C) according to the manufacturer’s instructions. In brief, either 200 μl of CM collected after 48 h of cell culture or 200 μl of a diluted IL-8 standard (31.25–1,000 pg/ml; six dilutions) was added per well (each sample was tested in triplicate) in high-binding, flat-bottomed, 96-well polypropylene plates (NUNC) precoated with IL-8 antibody (supplied in kit). After incubation at room temperature and sufficient wash with phosphate-buffered saline (PBS) + 1% Tween 20, the plate was treated with 200 μl of conjugate (supplied in kit) for 1 h at 2°C to 8°C, followed by addition of substrate in a dark condition at room temperature. Last, a stop solution (1 mol/L of sulfuric acid) was added in a volume of 50 μl to each well to stop the reaction. The absorbance readings at 450 nm (subtracted from 579-nm readings) were determined using a SpectraMax 250 microplate reader (Molecular Devices, San Jose, CA, United States). The concentration (in pg/ml) of IL-8 was converted from absorbance readings by using a standard curve generated from absorbance readings of standard samples. This assay was repeated three times along with negative controls.

### Treatment of HEY-A8 With Interleukin-8 and Cancer-Associated Fibroblast Conditioned Media

HEY-A8 was treated with IL-8 at the concentration of 200 ng/ml, or CAF CM alone, or CAF CM plus IL-8 antibody (10 ng/ml) over a time course of 24, 48, and 72 h. Cells were performed for Western blotting analysis.

### Three-Dimensional Culture of Ovarian Cancer Cells

For three-dimensional (3-D) culture of ovarian cancer cells, Rat Tail Collagen Type 1 (RTCT1, from BD Biosciences, San Jose, CA, United States) was used as matrigel and diluted to 50 μg/ml using 0.02 N of acetic acid; 24-well tissue culture plates were coated with matrigel at 5 μg/cm^2^ and allowed to solidify for 1 h at room temperature. Ovarian cancer cells were trypsinized from monolayer cultures and pelleted at 1,000 rpm. Then the cells were washed twice with PBS to remove cell debris and counted under a microscope. A total of 250 cells were resuspended in 1 ml of fresh media mixed with RTCT1 and NaOH at the ratio of 10:1:0.0235 and plated into each well of precoated 24-well plates. Cells were kept at room temperature for 10 min and cultured for 5–10 days at 37° with 5% CO_2_ in an incubator. 3-D spheroids were counted and recorded.

### Measurement of Stem-Cell Like Properties in Notch-Activated Cells From 3-D Culture

Cells expressing GFP with activated Notch signaling were sorted with flow cytometery and replated for 3-D culture in 24 wells at 250 cells/well. The number and size of spheroids were counted and examined in comparison with Notch-inactivated cells without expression of GFP.

### Analysis of Protein Expression by Immunoblotting

Total protein extract for each cell line was obtained by using a lysis buffer as described previously (Yang et al., 2010), and equal amounts (20 μg per load) were analyzed by immunoblotting. Antibody against β-actin was from Sigma-Aldrich (St. Louis, MO, United States) (A5441, 1:20,000); vascular endothelial growth factor (VEGF; sc-507, 1:1,000) was from Santa Cruz Biotechnology (Dallas, TX, United States). AKT (no. 9272, 1:1,000), extracellular signal-regulated kinase 1/2 (ERK1/2; mAb no. 4695, 1:1,000), and thrombospondin-1 (TSP-1) were from Lab Vision (MS-418, 1:500; Thermo Fisher Scientific, Waltham, MA, United States). Notch3 was from Cell Signaling Technology (Danvers, MA, United States) (cst-5276, 1:1,000). Western blotting reagents were from a chemiluminescence kit (Amersham Biosciences, Little Chalfont, United Kingdom).

### Animal Experiments

Animal experiments were approved by the Institutional Animal Care and Use Committee of Fudan University Shanghai Cancer Center (FUSCC) and performed following the Institutional Guidelines and Protocols. The 6- to 7-week-old BALB/c athymic nude mice were purchased from Shanghai Slac Laboratory Animal Co., Ltd. (Shanghai, China) and housed in the Department of Laboratory Animals (Fudan University). To test whether the CAFs enhance EOC growth, 3 × 10^6^ of tumor cells (HEY-A8) alone or mixed with 3 × 10^4^ (100:1) of differently treated fibroblasts in 150 μl of PBS were injected into the dorsal flap of one mouse. The date at which the first grossly visible tumor appeared was recorded, and tumor size was measured every 3–7 days thereafter. Tumor volume was measured and recorded according to our previous method (Yang et al., 2010). Statistical analysis was done by Fisher’s exact test at different time points for the mean tumor sizes of each group. When a tumor reached 1.5 cm in diameter, all mice in the same group were sacrificed by exposure to 5% carbon monoxide. The specific groups are as follows: each group including four to eight mice injected with 3 × 10^6^ cells for ovarian cancer cell line HEY-A8 from subcutaneous was considered as the control group.

### Immunohistochemical Staining

Ovarian normal and cancer tissues were obtained from FUSCC. Specimen collection was approved by the Clinical Research Ethics Committee of FUSCC and conducted with an informed consent signed by each participant before the use of tissues (No. 1711178-23). Tissue microarrays included core samples from 12 high-grade serous EOCs. Proteins including IL-8 and Notch3 were detected by immunostaining. Antibody was detected by using avidin–biotin–peroxidase method, as described elsewhere (Yang et al., 2010). Tissues with more than 5% of cells stained for the proteins were considered positive, and those with less than 5% staining were considered negative.

### Immunocytochemistry

The spheroids cultured in 3-D were harvested and washed with PBS for four times, then fixed in 75% ethanol for 2 h, and embedded in paraffin. The paraffin was cut into 3- to 5-μm-thick slides, deparaffinized in xylene, soaked in alcohol with different concentrations (100, 95, and 75%), and rinsed with deionized water. The slides were placed in 3% hydrogen peroxide in methanol and rinsed in water. The slides were steamed in the pretreatment buffer for 50 min and then cooled for 30 min. The slides were stained with Notch3 (1:100 dilution) and incubated overnight, and the polymer was added and incubated for 40 min. 3,3-Diaminobenzidine tetrahydrochloride was added and incubated for 20 min. The slides were counterstained with hematoxylin for 1 min.

### Measurement of Interleukin-8 in Ascites

The collection of ascetic fluid was performed in 12 EOC patients who were verified via histological analysis. The cell debris in ascitic fluid was removed by centrifugation at 2,500 rpm for 15 min. Each supernatant was collected, and then IL-8 was performed by the ELISA, as mentioned above.

### Statistical Analysis

The data were calculated using GraphPad Prism IV software and expressed as the mean ± SD. The difference between two groups was evaluated by Student’s *t*-test and chi-square test analyses. Values of *p* < 0.05 were considered statistically significant (^∗^*p* < 0.05; ^∗∗^*p* < 0.01; ^∗∗∗^*p* < 0.001). Center values are mean, and error bars are SD.

## Results

### Interleukin-8 Activates Normal Ovarian Fibroblasts in Multiple Signal Pathways

**FIGURE 1:**
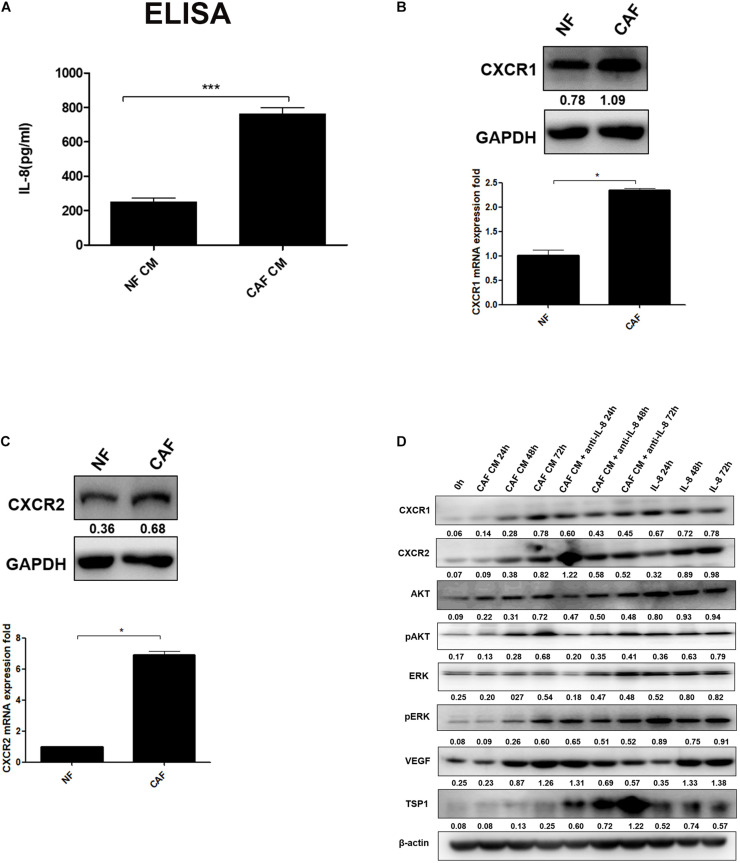
IL-8 activates NFs in multiple signal pathways. **(A)** Measurement of IL-8 in CAF CM and NF CM by ELISA. **(B,C)** Detection of CXCR1 and CXCR2 in NFs and CAFs by Western blotting and qRT-PCR. Triple independent experiments were carried out, and representative results are shown. **(D)** Analysis of AKT/ERK-associated proteins including CXCR1/2, AKT, pAKT, ERK, pERK, and angiogenesis-related proteins (VEGF and TSP-1) by Western blotting in NFs treated with CAF CM, CAF CM + anti-IL-8, and IL-8 alone. β-Actin was used as a loading control. *p < 0.05, ***p < 0.001. IL, interleukin; NFs, normal ovarian fibroblasts; CAFs, cancer-associated fibroblasts; CM, conditioned media; VEGF, vascular endothelial growth factor.

As a predominant cell type in normal stroma, quiescent resident fibroblasts can be transformed into CAFs through interaction with CAFs and/or cancer cells to become a major component of the tumor stroma, but little is known how NFs are transformed into CAFs (Mitra et al., 2012). In this study, we first isolated and successfully cultured the primary cultured CAFs and NFs from ovarian cancer and normal tissues, and then we measured the secretion of IL-8 in CAF CM and NF CM by ELISA. The results showed that the level of IL-8 was higher in CAF CM than NF CM (Figure 1A). It is generally known that the biological function of IL-8 is mediated through the binding of two cell-surface G protein-coupled receptors, CXCR1 and CXCR2, so their expression levels in NFs and CAFs were detected by Western blotting and qRT-PCR. We found that both receptors were highly expressed in CAFs compared with those in NFs (Figures 1B,C).

Furthermore, we treated NFs with CAF CM, CAF CM+IL-8 antibody (anti-IL-8), and IL-8 alone for 24, 48, and 72 h, respectively. As shown in Figure 1D, treatment of NFs with CAF CM increased the protein expression of CXCR1/2, AKT, pAKT, ERK, pERK, and VEGF in a time-dependent manner compared with control (0 h). Although treatment of NFs with IL-8 alone highly enhanced the expression of these molecules, the use of IL-8 antibody (anti-IL-8) to neutralize IL-8 in CAF CM only slightly reduced the expression of CXCR1/CXCR2, AKT, pAKT, ERK, pERK. The potential reasons are that more cytokines other than IL-8 from the CAF CM might also activate these signal molecules and that NFs also secreted IL-8 to rescue the neutralization during the treatment. However, neutralization of IL-8 with IL-8 antibody in NF treated with CAF CM apparently downregulated VEGF but upregulated thrombospondin 1 (TSP1), an anti-angiogenesis factor, in a time dependent manner. Therefore, it can be inferred that IL-8 activates NFs through PI3K/AKT and ERK pathways.

### Interleukin-8 Secreted From Cancer-Associated Fibroblasts Promotes Cancer Cell Stemness

**FIGURE 2:**
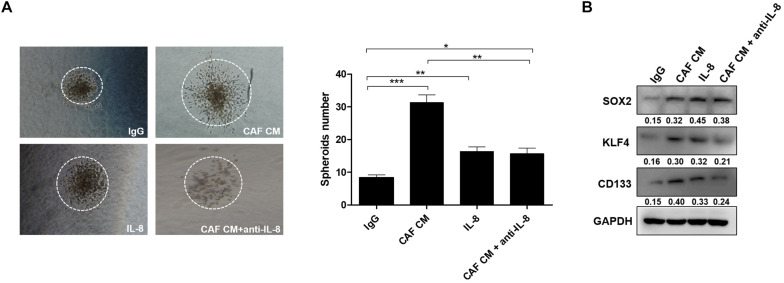
IL-8 secreted from CAFs promotes cancer cell stemness. **(A)** Spheroids formed by HEY-A8 cells treated with IgG, CAF CM, CAF CM+anti-IL-8, or IL-8. **(B)** Analysis of KLF4, SOX2, and CD133 expression by Western blotting in HEY-A8 cells treated with IgG, CAF CM, CAF CM+anti-IL-8, or IL-8. GAPDH was used as a loading control. IL, interleukin; CAFs, cancer-associated fibroblasts. *p < 0.05, **p < 0.01, ***p < 0.001.

Since CSCs play a critical role in the formation of spheroids, we explored whether IL-8 could maintain tumor stemness through regulation of cancer cell growth and invasion. As the results shown in 3-D culture, the spheroids formed by cancer cells treated with CAF CM developed bigger than those formed by the control cells treated with IgG, or spheroids treated with IL-8 alone and CAF CM+anti-IL-8. Meanwhile, spheroids of cancer cells treated with IL-8 were much bigger than those of cells group treated with CAF CM+anti-IL-8, indicating that IL-8 derived from CAFs may promote proliferation and stemness of cancer cells. The number of spheroids formed by cells treated with CAF CM was significantly more than that formed by the cells treated with others (Figure 2A).

Further experiments showed that the stemness markers including KLF4, SOX2, and CD133 were increased by IL-8 stimulation, suggesting that IL-8 secreted from CAFs may promote ovarian cancer cell stemness (Figure 2B).

### Interleukin-8 Stimulates Xenograft Tumor Growth in Animals

**FIGURE 3:**
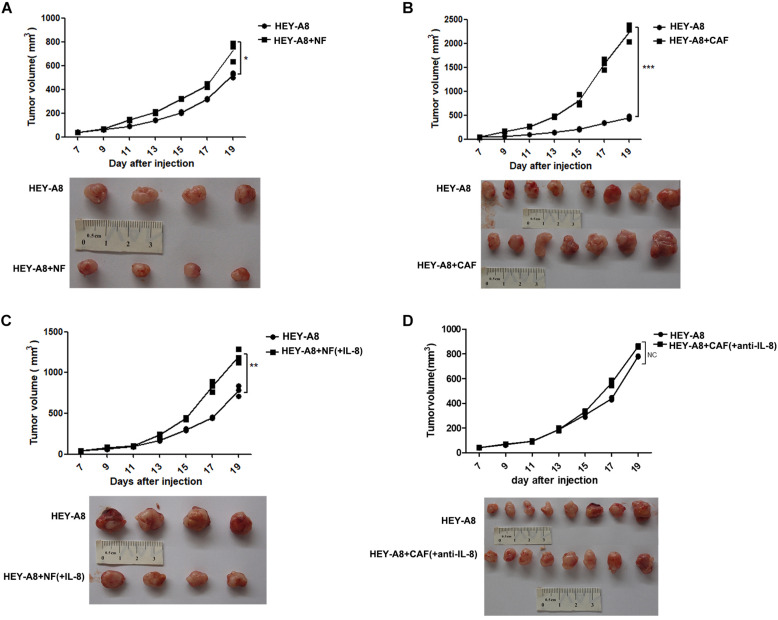
IL-8 stimulates xenograft tumor growth in animals. Xenograft tumor growth by subcutaneous injection of HEY-A8 cells mixed with different fibroblast NFs **(A)**, CAFs **(B)**, NFs+IL-8 **(C)**, or CAFs neutralized by IL-8 antibody **(D)** as indicated. *p < 0.05, **p < 0.01, ***p < 0.001. IL, interleukin; NFs, normal ovarian fibroblasts; CAFs, cancer-associated fibroblasts.

To investigate whether CAFs promote the growth of tumor *in vivo*, we injected ovarian cancer epithelial cells alone or with CAFs, and NFs treated with or without IL-8 into immunocompromised mice. The results showed that HEY-A8 cells mixed with NFs enhanced cancer growth as compared with HEY-A8 cells alone (Figure 3A), whereas HEY-A8 cells mixed with CAFs stimulated tumor even much faster than cancer cells alone (Figure 3B). Although NFs+IL-8 induced the tumor growth of HEY-A8 cells faster than did HEY-A8 cells alone, cancer cells pretreated with CAF CM+IL-8 antibody to neutralize the function of IL-8, generated the similar tumor growth to that induced by HEY-A8 cells alone (Figures 3C,D).

### Interleukin-8 Enhances Ovarian Cancer Cell Stemness Through Notch3 Signaling Pathway

**FIGURE 4:**
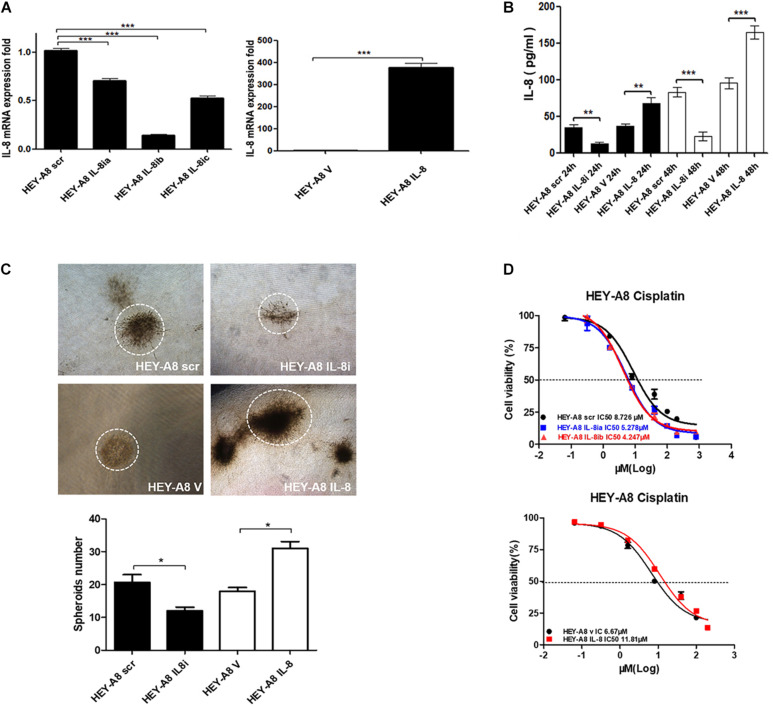
IL-8 enhances ovarian cancer cell stemness and chemoresistance. **(A)** Examination of IL-8 expression in IL-8 overexpression and silencing ovarian cancer cells by qRT-PCR. **(B)** Measurement of IL-8 secreted from overexpression and silencing cells by ELISA. **(C)** Spheroids formed by IL-8 overexpression and silencing ovarian cancer cells in 3-D culture. **(D)** Survival rates of ovarian cancer cells expressing IL-8 shRNA or cDNA tested by CCK-8 following the treatment with cisplatin. OD values were measured after treatment with cisplatin at 48 h. *p < 0.05, **p < 0.01, ***p < 0.001. IL, interleukin; CCK-8, Cell Counting Kit-8; OD, optical density.

As a chemokine, IL-8 can be secreted into the tumor microenvironment by cancer cells in an autocrine manner to trigger a series of biological functions through the surface receptors in cancer cells. To understand the effect of IL-8 on cancer cells, IL-8 cDNA and shRNA were, respectively, introduced to HEY-A8 cells. qRT-PCR was used to verify that IL-8 was remarkably overexpressed or silenced in the resulting cells compared with control cells treated with empty vector (V) or scrambled shRNA (scr). From the data, we selected the cells expressing IL-8ib in the next experiment (Figure 4A). The secreted levels of IL-8 in both two cell lines were measured by ELISA. Compared with that of the control, the level of secreted IL-8 was significantly decreased in HEY-A8 IL-8i cells and increased in HEY-A8 IL-8 cells (Figure 4B).

The 3-D approach has been used in cancer research as an intermediate model between *in vitro* cancer cell line cultures and *in vivo* tumors, formation has gained popularity in CSC research (Weiswald et al., 2015). Thus, we confirmed proliferation and stemness of cell lines by 3-D culture. As shown in Figure 4C, the spheroids formed by HEY-A8 IL-8i cells were smaller than those formed by control cells. The spheroids formed by HEY-A8 IL-8 cells developed much bigger than those formed by control cells.

Chemotherapeutic resistance, whether intrinsic or acquired, is a multifactorial phenomenon that is associated with the tumor microenvironment (Sui et al., 2013). To explore the impact of IL-8 on chemoresistance, we evaluated the effects of IL-8 on response of cancer cells to cisplatin (CDDP). Cells treated with different concentrations of CDDP at 48 h were determined by Cell Counting Kit-8 (CCK-8) assay for their viabilities. Compared with that in control cells, the survival rate was increased in overexpressing cells, whereas that of cells expressing IL-8 shRNA was decreased. The IC_50_ values of CDDP were increased in cells overexpressing IL-8. In contrast, the IC_50_ values of IL-8 knockdown cells were reduced compared with those of control cells (Figure 4D). These data suggest that IL-8 confers CDDP resistance in ovarian cancer cells.

**FIGURE 5:**
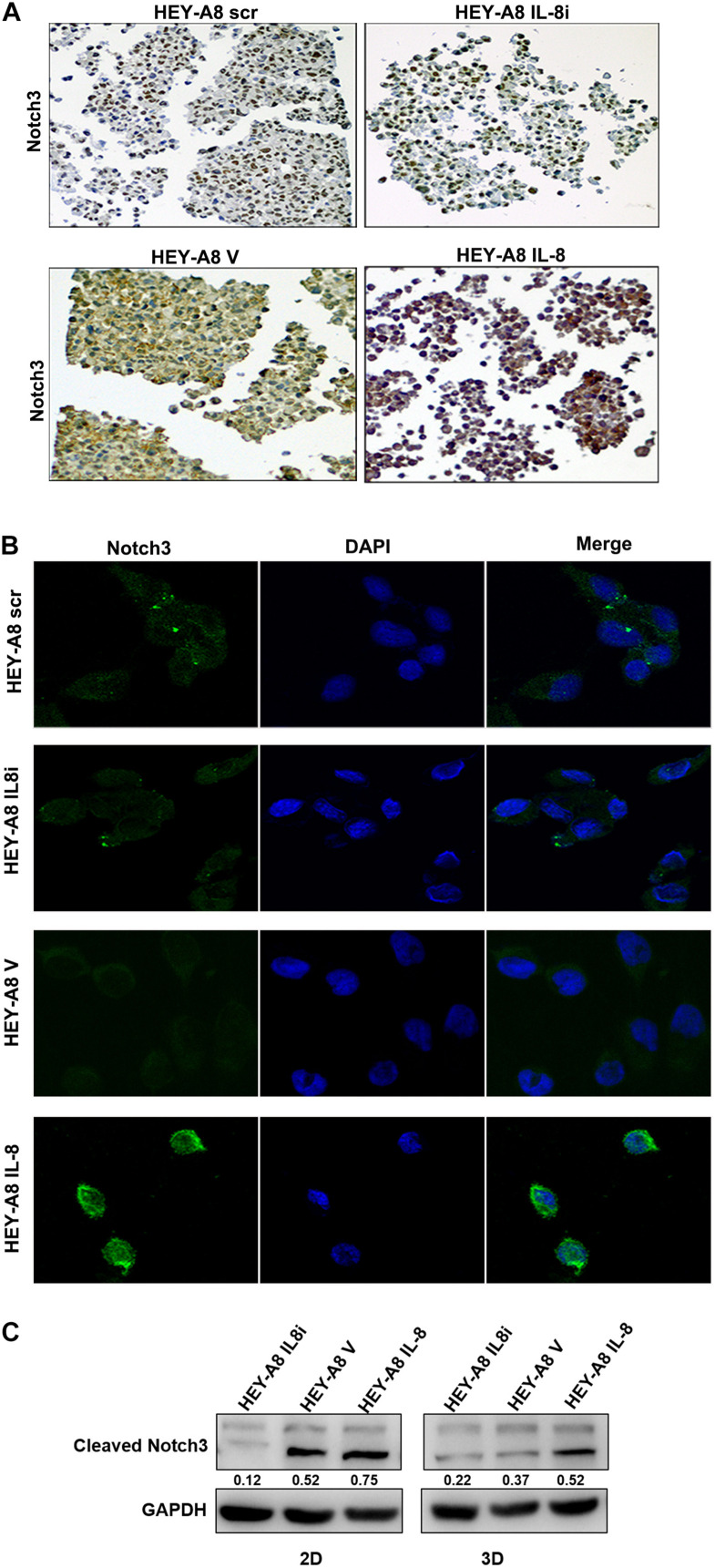
IL-8 enhances ovarian cancer cell stemness through the Notch3-mediated signaling pathway. **(A)** Examination of Notch3 expression in spheroids by immunocytochemistry. **(B)** Notch3 activation in cells derived from spheroids. Notch3 was activated (green) in 3-D culture cells with IL-8 overexpression but was decreased in IL-8-silencing cells compared with control cells. DAPI (blue) was used as a nuclear dye. **(C)** Analysis of cleaved Notch3 by Western blotting in HEY-A8 cells cultured in 2-D and 3-D. IL, interleukin.

It has been reported that the Notch family is important for stem cell self-renewal through the enhanced cancer cell stemness in CSCs (Takebe et al., 2015). The Notch signaling can be activated by factors such as IL-6, which derived from CAFs (Guo et al., 2011). We then further explored the relationship between Notch3 and IL-8. 3-D spheroids were analyzed by immunochemical staining and immunofluorescence and showed that the expression of Notch3 was obviously higher in HEY-A8 IL-8 cells and lower in HEY-A8 IL-8i cells than in control cells (Figures 5A,B).

To further investigate whether Notch3 is involved in pathway of IL-8-mediated tumor stemness, Western blotting was used to detect the expression of Notch3 in IL-8 knockdown and overexpression cells derived from 2-D and 3-D culture. The results showed that the cleaved Notch3 was decreased in IL-8 knockdown cells but was increased in IL-8 overexpression cells, indicating that IL-8 may promote ovarian cancer cell stemness through the activation of Notch3 signaling (Figure 5C). However, the expression of Nocth1 and Notch2 did not obviously change between cells derived from the 2-D and 3-D cultures (Supplementary Figure 1).

### Expression of Interleukin-8 Is Associated With Notch3 in Ovarian Cancer Tissues

**FIGURE 6:**
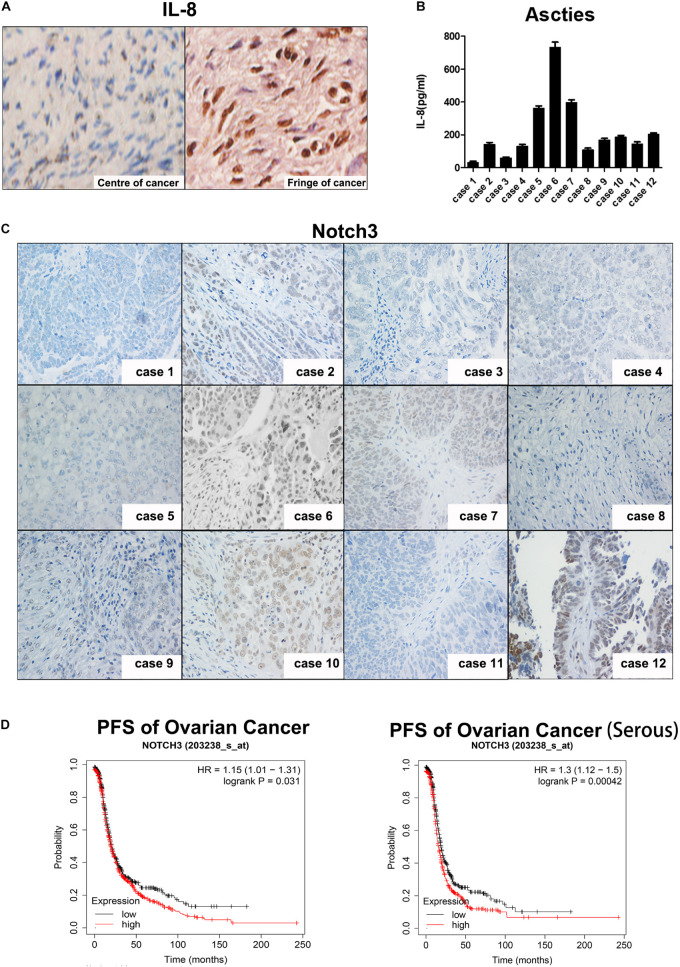
Expression of IL-8 is associated with Notch3 in ovarian cancer tissues. **(A)** IHC staining of IL-8 expression in ovarian cancer tissues. Center (left) and fringe (right). Magnification, ×400. **(B)** IL-8 levels in ascitic fluid of 12 EOC patients were measured by ELISA. **(C)** Representative images showing Notch3 expression detected by IHC in ovarian cancer tissues. Magnification, ×200. **(D)** Prognostic analyses of ovarian cancer patients based on Notch3 mRNA levels calculated by the Kaplan–Meier survival curves (http://kmplot.com/analysis/index.php?p=service). IL, interleukin; IHC, immunohistochemistry; EOC, epithelial ovarian cancer.

To determine whether IL-8 is expressed in tumor tissues in patients and which cells IL-8 is expressed in, we performed immunohistochemistry. As shown in Figure 6A, IL-8 was highly expressed in the stroma of ovarian tumor fringe tissues, where there were more CAFs (right), but little IL-8 was stained in the center of tissues with fewer fibroblasts (left).

We then investigated the relationship between Notch3 and IL-8 levels in patients diagnosed with ovarian cancer by examining the level of IL-8 in the ascites by ELISA and the expression of Nocth3 in tumor tissue samples from 12 ovarian cancer patients by immunohistochemical staining (Figures 6B,C). Interestingly, the expression of IL-8 in ascites was positively correlated with the nuclear expression of Notch3 in tissues.

Finally, we explored the critical role of Notch3 in the survival of patients with ovarian cancer. The Kaplan–Meier curve and log rank test analyses revealed that the high expression of Notch3 mRNA had a lower progression-free survival (PFS) rate in all pathological types of ovarian cancer, especially in serous ovarian cancer (Figure 6D).

## Discussion

**FIGURE 7:**
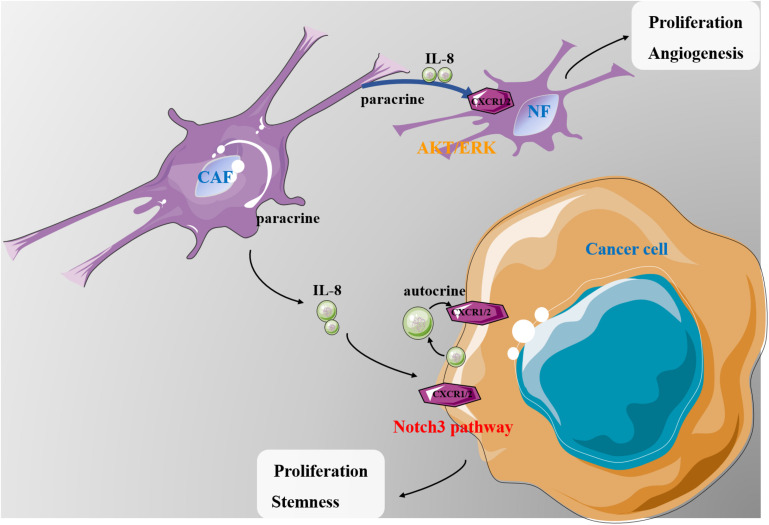
A schematic diagram showing the possible mechanism of IL-8 in ovarian cancer microenvironment. CAF-derived IL-8 can activate the NFs in multiple signal pathways. IL-8 derived from both cancer cells and CAFs can promote proliferation and stemness of cancer cells through the Notch3-mediated signaling. IL, interleukin; CAFs, cancer-associated fibroblasts; NFs, normal ovarian fibroblasts.

In this study, we provide strong evidence that IL-8 plays a critical role in ovarian cancer microenvironment (Figure 7). CAFs have been reported to possess a prominent role in promoting tumor growth and predicting poor outcome in various cancers, which have gained many attentions as a promising target (Erdogan and Webb, 2017). CAF CM contains a bunch of growth factors, cytokines and chemokines, which may promote proliferation and survival of cancer cells (Nurmik et al., 2020). IL-8 is one of the critical chemokines in CAF CM. In the present study, we found that a higher level of IL-8 was present in CAF CM than in NF CM. Mechanistic studies revealed that IL-8 could activate NFs through PI3K/AKT and ERK signals. Furthermore, IL-8 also stimulated angiogenesis by increasing VEGF and decreasing TSP1 in NFs. The *in vivo* experiments showed that cancer cells mixed with NFs treated with IL-8 promoted ovarian xenograft tumor growth in mice. In 3-D culture, IL-8 induced the spheroid formation of ovarian cancer cells, whereas the IL-8-mediated cell stemness might be mediated through the Notch3 signaling. Analysis of clinical tissues further revealed that the levels of IL-8 in patient ascites were closely correlated with the activation of Notch3, suggesting a close link between IL-8 and Notch3, although the detailed mechanism requires further investigations.

Many cancers appear to contain a small population of pluripotent “CSCs” (Al-Hajj et al., 2004). The CSC hypothesis states that CSCs possess some of the biological properties of normal stem cells, including indefinite self-replication, asymmetric cell division, and resistance to toxic agents (De Francesco et al., 2018; Najafi et al., 2019). The CSC proliferation rate may depend on cancer microenvironment and also be associated with signal pathways (Eun et al., 2017). CSCs are responsible for chemotherapeutic resistance and finally cause disease recurrences and/or metastasis (Chang, 2016; Das et al., 2020). Ovarian cancer cell line HEY-A8 is reported to be enriched with CSCs. The Notch signaling members are critical in the regulation of CSCs, among which Notch3 overexpression in cancer cells can result in expansion of CSCs and increase platinum resistance (McAuliffe et al., 2012). In our study, we also found that overexpression of IL-8 in HEY-A8 can enhance CDDP resistance. However, whether IL-8 mediates chemoresistance via Notch3 remains unknown. Based on literature, the AKT pathway has been reported to stimulate the Notch activity in some experimental models, while VEGF is also found to regulate the expression of Notch genes through the phosphatidylinositol 3-kinase/AKT pathway (Liu et al., 2003). In the stromal microenvironment, many chemokines and cytokines may activate PI3K/AKT and Notch pathways, leading to tumor initiation and development, so we speculate that IL-8 may maintain the stemness of tumor cells through the PI3K/AKT/Notch3 pathway.

Certainly, there were still some limitations in our study. We only focused on the IL-8 derived from CAFs through which to activate NFs. However, in Figure 1, we also found that when NFs were treated with CAF CM and anti-IL-8, the expression of CXCR1/2, VEGF, and pERK was also increased, which prompted us to think whether other cytokines in CAF CM cooperated with IL-8 to promote the activation of NFs. CXCR1 and CXCR2 are reported to promote proliferation and invasion in many cancer cells. However, whether CAF-derived IL-8 mediates stemness via CXCR1/2 remains unknown.

Our data show that in ovarian tumor microenvironment, IL-8 derived from cancer cells and CAFs promotes the proliferation and stemness of cancer cells through the Notch3 signaling pathway, suggesting that the IL-8/Notch3 signaling may be a potential target for ovarian cancer treatment in the future.

## Data Availability Statement

The original contributions presented in the study are included in the article/Supplementary Material, further inquiries can be directed to the corresponding author/s.

## Ethics Statement

The studies involving human participants were reviewed and approved by the Clinical Research Ethics Committee of Fudan University Shanghai Cancer Center. Written informed consent to participate in this study was provided by the participants’ legal guardian/next of kin. The animal study was reviewed and approved by the Institutional Animal Care and Use Committee of Fudan University Shanghai Cancer Center.

## Author Contributions

ZJ, WT, and WG performed the experiments, collected all data, and drafted the manuscript. RZ and HW participated in the project design and manuscript discussion. GY designed the project and edited the manuscript. All authors contributed to the article and approved the submitted version.

## Conflict of Interest

The authors declare that the research was conducted in the absence of any commercial or financial relationships that could be construed as a potential conflict of interest.
