# Supplementary material for: Cancer-Associated Fibroblast-Derived Interleukin-8 Promotes Ovarian Cancer Cell Stemness and Malignancy Through the Notch3-Mediated Signaling
Source: Front Cell Dev Biol. 2021 Jul 1;9:684505. doi: 10.3389/fcell.2021.684505 (PMC8280773; doi:10.3389/fcell.2021.684505)
Supplement: Supplementary file 1 [file Data_Sheet_1.PDF]

Supplementary figure

Analysis of Noch1 and Notch 2 expression in HEY-A8 cells cultured in 2-D and 3-D by western blot. The IL-8 overexpression and silencing cells were cultured in 2-D and 3-D culture separately, and then detect the expression of Noch1 and Notch 2 by western blot.
