# Supplementary figures and images for: Cancer-Associated Fibroblast-Derived Interleukin-8 Promotes Ovarian Cancer Cell Stemness and Malignancy Through the Notch3-Mediated Signaling
Source: Front Cell Dev Biol. 2021 Jul 1;9:684505. doi: 10.3389/fcell.2021.684505 (PMC8280773; doi:10.3389/fcell.2021.684505)

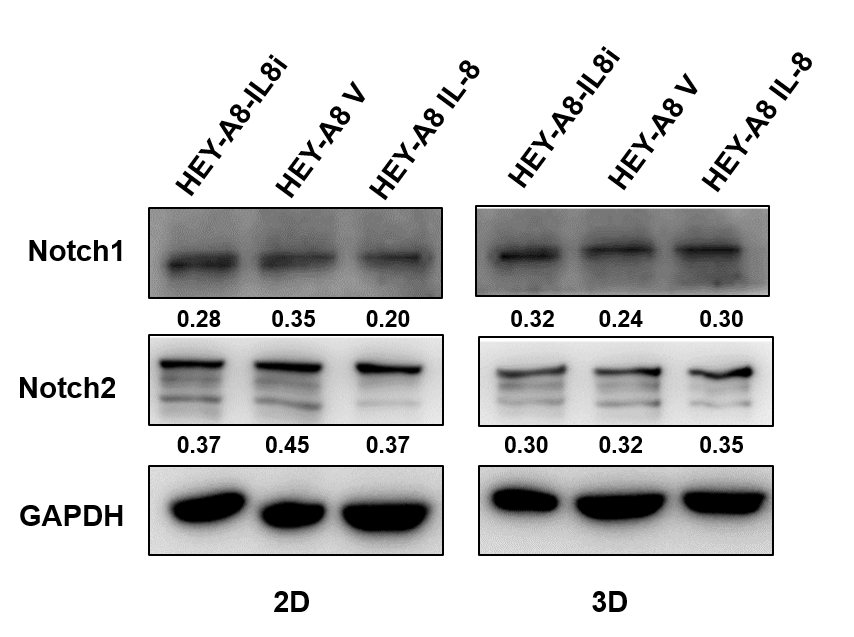

Supplement: Supplementary file 2 [file Image_1.TIF]
