# Supplementary material for: Cancer-Associated Fibroblast-Derived Interleukin-8 Promotes Ovarian Cancer Cell Stemness and Malignancy Through the Notch3-Mediated Signaling
Source: Front Cell Dev Biol. 2021 Jul 1;9:684505. doi: 10.3389/fcell.2021.684505 (PMC8280773; doi:10.3389/fcell.2021.684505)
Supplement: Supplementary file 3 [file Table_1.pdf]

Supplementary table

Sequences of primers are provided in **table1**

**Gene**	**5-3 primer sequence**	
IL-8	Forward: CTCTTGGCAGCCTTCCTGATTTC<br>Reverse: TTTTCCTTGGGGTCCAGACAGAG	qRT-PCR
CXCR1	Forward: CTGACCCAGAAGCGTCACTTG<br>Reverse: CCAGGACCTCATAGCAAACTG	
CXCR2	Forward: CCTGTCTTACTTTTCCGAAGGAC<br>Reverse: TTGCTGTATTGTTGCCCATGT	
IL-8-sh1	Forward: CCGGCAAGGAGTGCTAAAGAACTTACTCGAGTAAGTTCTTTAGCACTCCTTGTTTTTG<br>Reverse: : AATTCAAAAACAAGGAGTGCTAAAGAACTTACTCGAGTAAGTTCTTTAGCACTCCTTG	sh
IL-8-sh2	Forward: CCGGGCTCTGTGTGAAGGTGCAGTTCTCGAGAACTGCACCTTCACACAGAGCTTTTTG<br>Reverse: AATTCAAAAAGCTCTGTGTGAAGGTGCAGTTCTCGAGAACTGCACCTTCACACAGAGC	
IL-8-sh3	Forward: CCGGACTTAGATGTCAGTGCATAAACTCGAGTTTATGCACTGACATCTAAGTTTTTTG<br>Reverse: AATTCAAAAAACTTAGATGTCAGTGCATAAACTCGAGTTTATGCACTGACATCTAAGT	
